# Do Internet Search Data Help Forecast Air Passenger Demand? Evidence From China’s Airports

**DOI:** 10.3389/fpsyg.2022.809954

**Published:** 2022-06-16

**Authors:** Xiaozhen Liang, Qing Zhang, Chenxi Hong, Weining Niu, Mingge Yang

**Affiliations:** ^1^School of Management, Shanghai University, Shanghai, China; ^2^Business School, Beijing International Studies University, Beijing, China

**Keywords:** air passenger demand, Internet search data, multivariate empirical mode decomposition, deep learning algorithm, time series analysis

## Abstract

Before making travel plans, people often use the Internet to collect relevant information to help themselves make better decisions. Among the numerous information search channels, Internet search engine is used by the vast number of travelers because of its low cost and high efficiency. To a large extent, Internet search behavior is the external manifestation of users’ psychological activities, reflecting their concerns, needs and preferences. Therefore, Internet search data can reflect the air passenger demand information to a certain extent. In this manuscript, a novel decomposition ensemble model is proposed to discuss the role of Internet search data in air passenger demand forecasting. In the empirical study, the relevant data of Shanghai Pudong International Airport and Beijing Capital International Airport are taken as samples. The results show that the proposed forecasting model can integrate the advantages of decomposition-ensemble strategy and deep learning algorithm, and achieve more accurate and reliable prediction results than all benchmark models. This further indicates that adding Internet search data into the forecasting model can effectively improve the prediction performance of air passenger demand, and can provide scientific and reliable decision support for air transport management.

## Introduction

In the global scope, the frequent trade activities between countries and regions and the rapid development of tourism industry have led to the development of various transport modes. In particular, air travel has become more and more popular in middle-distance and long-distance tourism in recent years due to its safety, comfort, convenience and acceptable fares, which has greatly promoted the development of air transport industry. According to statistics from the Civil Aviation Administration of China (CAAC), in 2019, China’s air passenger turnover reached 1,170.51 billion passenger-kilometers, up 9.3% year on year. Meanwhile, China’s civil aviation airports handled a passenger throughput of 1.352 billion, up 6.9% year on year. The increasing air passenger demand poses challenges to air transport management. Therefore, it is of great significance to accurately forecast air passenger demand, which is helpful for the rapid and efficient management of the air transport industry, and can provide reference for the scientific and effective decision-making of relevant government departments and civil aviation enterprises (Xie et al., 2014).

In the past decades, scholars have done a lot of research on air passenger demand forecasting. However, the previous studies are almost based on the statistical data that are drawn from the historical observation records, with traditional data sources. These studies use historical data to predict the future, which cannot accurately reflect the uncertainty of the future and cannot help predict the future mutations. Hence the prediction result is not accurate enough for the prediction of structural changes. With the rapid development and increasing popularity of the Internet, people tend to use the Internet to collect relevant information before making travel plans and then help themselves make better decisions. Given the important role of sensory cues in consumption (Wang W. et al., 2022), it is worth studying whether the rich Internet search data can be applied to the field of air passenger demand prediction to make up for the deficiency of using only historical statistical data. However, few studies have discussed the role of Internet search data in air passenger demand forecasting. There are several issues worthy of in-depth analysis and discussion in practical application, such as whether there is a relationship between Internet search behavior and air passenger demand, how to choose the appropriate Internet search data, and how to establish a high-precision model for air passenger demand forecasting accordingly.

In addition, the air passenger demand data is seasonal, random and nonlinear, which makes the simple models commonly used in the past can only extract a small amount of information. In recent years, deep learning models have been widely used in the field of prediction due to their excellent ability to capture nonlinear sequence features, which gives us some enlightenment. Meanwhile, as the prediction results of single models are not stable, scholars tend to use hybrid models for prediction, among which decomposition-ensemble prediction is the current mainstream strategy. Previous studies have shown that the prediction performance of the model can be significantly improved by using the “decomposition-ensemble prediction” strategy, which provides a reference for air passenger demand forecasting.

In this manuscript, we aim to build a hybrid forecasting model combining Internet search data and deep learning algorithms. The main contributions of this manuscript include: (1) We introduce the Internet search data into the forecast of air passenger demand, and propose a method for screening Internet search data based on the maximum information coefficient (MIC) analysis. The empirical results show that the screened Internet search data is helpful to improve the forecast accuracy of air passenger demand; (2) Based on the “decomposition-ensemble prediction” strategy and deep learning algorithm, this manuscript establishes a novel forecasting framework for air passenger demand considering the Internet search data, which can fully extract the data features and obtain accurate prediction results.

The rest of this manuscript is organized as follows. “Literature Review” section provides an academic foundation. “Correlation Mechanism Analysis” section analyzes the correlation mechanism between Internet search behavior and air passenger demand. “Materials and Methods” section describes the overall framework of the proposed model and the basic principle of the algorithms used in this manuscript. In “Results” section, an empirical analysis is conducted to verify the prediction performance of the proposed model. Then, the results are discussed in “Discussion” section. Finally, “Conclusion” section summarizes the manuscript with conclusions.

## Literature Review

Prosperity of big data has brought new vitality to different academic fields, such as mental health research (Hassani et al., 2022; Liang et al., 2022), buying behavior research (Ma and Liao, 2022; Wang J. et al., 2022), and so on. Among them, Internet search data is widely used with the advantage of easy access. There is no doubt that web search is a technology that almost everyone is familiar with and willing to use nowadays. More specifically, among the numerous information search channels, Internet search engine is used by the vast number of travelers because of its low cost and high efficiency. To a large extent, Internet search, a behavior that can alleviate the harm of knowledge hiding (Zhao et al., 2019), is the external manifestation of users’ psychological activities, reflecting their concerns, needs, and preferences.

In recent years, to make up for the deficiency of using only traditional data for prediction and capture the latest trend better, more and more scholars have applied Internet search data to prediction. Sun et al. (2019) proposed a prediction framework combining the KELM method and Internet search data, and based on the prediction experiment of Beijing tourist volume, concluded that adding Internet search data into the prediction model could significantly improve the prediction performance. Tang et al. (2020) proposed a multi-scale prediction model considering Internet search data for oil price, aiming at the different influences of Internet search data on oil price at different time scales. The empirical results show that the proposed model has better prediction performance than the benchmark models. Zhang et al. (2020) adopted Baidu search index to reflect investors’ concerns, thus establishing a novel volatility prediction model considering investors’ concerns. The empirical analysis shows that investors’ concerns reflected by Internet search data have a strong ability to explain and predict the stock market. In these studies, it was found that the performance of the prediction models showed significant improvement with the addition of Internet search data. However, it is necessary to further explore the method of applying Internet search data to air passenger demand forecasting.

The development of accurate air passenger demand forecasting models is a critical issue, which plays a positive role in promoting progress in international air transport industry. To this end, many such single models have been proposed. They can be broadly divided into two categories, i.e., econometric models (such as SARIMA, Holt-Winters, VAR, etc.) (Fildes et al., 2011; Tsui et al., 2014; Xu et al., 2019) and artificial intelligence models (such as SVR, KELM, etc.) (Liang et al., 2017; Jin et al., 2020). In the last decade, with the continuous improvement of computer performance, artificial intelligence models have been rapidly developed and widely applied in various fields, among which deep learning algorithms have been widely used in prediction research due to their unique advantages in data mining and analysis. For example, Kulshrestha et al. (2020) proposed a deep learning model named Bayesian bidirectional long short-term memory neural network (BBiLSTM), and conducted an empirical research with the tourism dataset of Singapore as a sample. The results show that the prediction effect of the proposed model is better than that of the benchmark models. Shen et al. (2020) combined the LSTM network with convolutional neural network (CNN) to form a dynamic time series prediction model, and proved that the proposed model has high prediction accuracy and robustness. Nevertheless, how to apply deep learning algorithms to air passenger demand forecasting remains to be further studied.

Although deep learning prediction models fill in many shortcomings of traditional prediction models, a single model can always fail to meet the high requirements of air passenger demand series prediction. Based on this, the “decomposition-ensemble prediction” strategy has gradually become an effective and satisfactory method for the scholars in the field of prediction. In general, the basic idea of this strategy is to decompose the original series into several components at the first step, and then model and predict each component, respectively. Finally, the prediction results of each component are integrated to obtain the final predicted values. Many prediction studies have fully demonstrated the effectiveness of this strategy and provided a lot of empirical reference for air passenger demand forecasting. For instance, Li et al. (2022) constructed a decomposition and integration model of ship motion prediction based on empirical mode decomposition (EMD) algorithm, and the empirical results proved the superiority of the proposed model. Huang et al. (2021) proposed a prediction model combining EMD and gated recurrent unit neural network (EMD-GRU) for non-stationary sequences (PM 2.5 concentration), and the results showed that the error of EMD-GRU model was significantly reduced compared with the single GRU model. Based on the above analysis, this manuscript proposes a novel decomposition-ensemble model based on Internet search data and deep learning algorithm, to discuss the role of Internet search data in air passenger demand forecasting.

## Correlation Mechanism Analysis

As an important channel for people to obtain information in daily life, search engine can generate Internet search data, which objectively and exhaustively record users’ search behaviors and the searched information, making it possible to study the rules of human psychology and behavior with the help of digital trace data from search engines (Wilson et al., 2012). Internet search data perform well in reflecting what people pay attention to, so they are applicable to predict the behaviors of the general public (Lai et al., 2017).

**FIGURE 1:**
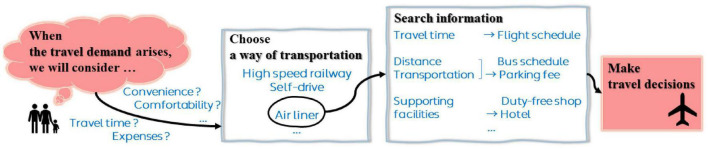
Correlation mechanism between information search and travel decision-making.

People are significantly affected by the social environment around them (Li et al., 2018), and gradually choose consumption patterns more rationally and actively under the influence of the development of the Internet (Yang et al., 2022). When the travel demand arises, people tend to collect relevant information from the Internet, including travel time, expenses, convenience, comfortability and so on, to help them make better travel plans. Figure 1 shows the correlation mechanism between Internet search behavior and travel decision-making. When considering whether to choose an airport as a destination, the search area is narrowed down to the information related to the airport, such as its flights, the distance and mode of transportation between the airport and urban areas and surrounding cities, hotels around the airport, and duty-free shops at the airport, etc. To a certain extent, this information contributes to the final decisions of the travelers, which will be further reflected in the changes of airport passenger flow. Among the numerous information search channels, Internet search engine is adopted by the vast number of travelers because of its low cost and high efficiency. Each search by the user will be recorded by the search engine, and the search index of the search term will be generated based on the search volume in days. The search index will perform as a recorder of users’ emotions, psychology and behavior and a tool to predict their travel decisions. Therefore, search engine data can reflect part of the air passenger demand information, which has a certain explanatory ability for air passenger demand forecasting.

However, search engine data often show the current trends of people’s demand and concerns about something, while traditional statistical data are better at reflecting long-term trends. Therefore, using search engine data alone to predict air passenger demand may not be very appropriate. Better prediction effect can be achieved by combining these two kinds of data to build a prediction model.

## Materials and Methods

### Multivariate Empirical Mode Decomposition

Empirical mode decomposition (EMD) is a technique to decompose nonlinear and non-stationary sequences into several intrinsic mode functions (IMFs) and a residual sequence, which has been widely used because of its obvious advantages in terms of self-adaptation. All signals can extract several IMF components with the frequencies from high to low in turn. That is to say, the IMF_1_ component extracted first has the highest frequency, and the IMF_*n*_ component extracted last has the lowest frequency (n is the number of IMF components). Physically, if the instantaneous frequencies are to be meaningful, then the corresponding IMFs must be symmetric, with a local mean of zero, and with the same number of zero crossing points and extreme points. However, EMD can only deal with 1-dimensional data. In order to process multi-dimensional data that often appears in the real-world, an emerging technique named multivariate empirical mode decomposition (MEMD) was proposed by Rehman and Mandic (2010). In this method, multi-dimensional envelopes can be obtained by projection operation of the multivariate signals. Then, we can get the local mean estimation of the multivariate signals through an averaging calculation. Furthermore, following the standard EMD procedure, the multivariate signals are decomposed into a set of multivariate sub-sequences from high frequency to low frequency (IMFis, i = 1, 2, 3, …) and multivariate residuals (Rs). The detailed steps of MEMD algorithm are as follows (Tang et al., 2020):

(1) Calculate the direction vectors *x*_θ*k*_(*k* = 1,…,*K*), Where *K* is the number of direction vectors.
(2) Get the projections *p*_θ*k*_(*t*) of multivariate signals *s*(*t*) along the direction vectors *x*_θ*k*_.
(3) Find the maximum value of the projection signal *p*_θ*k*_(*t*) corresponding to the time node $\left\{t_{\theta \,k}^{i}\right\}$.
(4) Use the method of interpolation to achieve the multivariate envelope curves *e*_θ*k*_(*t*) based on point $\left\{t_{\theta \,t}^{i},s\,\left(t_{\theta \,t}^{i}\right)\right\}$.
(5) Calculate the mean of the envelope curves $m\,\left(t\right)=\frac{1}{K}\,\sum_{k=1}^{K}e_{\theta \,k}\,\left(t\right)$.
(6) Extract the remaining ingredients *h*(*t*) = *s*(*t*)−*m*(*t*). If *h*(*t*) meets the criteria for the multiple IMF, defined as an IMF. If else, it will be repeated as the original multiple sequence in steps (2) to (5) until surface a *h*(*t*) meets the conditions of IMF.
(7) Repeat steps (2) to (6) with the remainder *r*(*t*) = *s*(*t*)−*h*(*t*) as the original signal until the IMF cannot be disassembled or certain ending-conditions are met.

Finally, MEMD decomposes multivariate data into several IMF groups and a residual group, with each channel containing the same number of IMFs. Moreover, it can effectively identify the common factors from interrelated multivariate data at similar timescales. Using univariate EMD to decompose each single sequence separately cannot achieve such a strict mode alignment.

### Bidirectional Long Short-Term Memory

**FIGURE 2:**
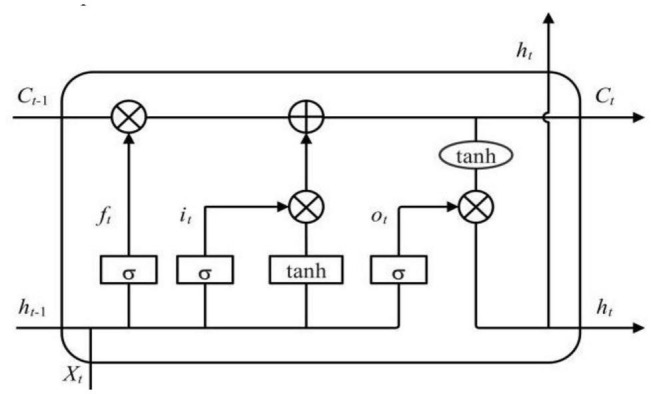
Structure of an long short-term memory (LSTM) memory cell.

Long short-term memory (LSTM) network is a modified version of recurrent neural network (RNN), which is used to solve the problem about the long-term dependence of data (Kulshrestha et al., 2020). A typical LSTM consists of an input layer, an output layer, and one or more hidden layers, and the hidden layer is composed of multiple memory cells. As shown in Figure 2, each memory cell contains three gates including an input gate *i*_*t*_, a forget gate *f*_*t*_ and an output gate *o*_*t*_, which can selectively retain or forget the relevant contextual information (Du et al., 2020). The input gate is used to control the input of information, the forget gate determines how much information should be transferred to the next cell, and the output gate is used to control the output of the information. Because of the existence of these gate structures, LSTM can make information affect the state of each moment selectively. The following equations are used to express LSTM:


(1) $$f_{t}=\sigma \,\left(W_{f}\cdot \left[h_{t-1},X_{t}\right]+b_{f}\right)$$

(2) $$i_{t}=\sigma \,\left(W_{i}\cdot \left[h_{t-1},X_{t}\right]+b_{i}\right)$$

(3) $$o_{t}=\sigma \,\left(W_{o}\cdot \left[h_{t-1},X_{t}\right]+b_{o}\right)$$

(4) $$C_{t}=f_{t}⊙C_{t-1}+i_{t}⊙\tanh\left(W_{C}\cdot \left[h_{t-1},X_{t}\right]+b_{C}\right)$$

(5) $$h_{t}=o_{t}⊙\tanh\left(C_{t}\right)$$

where *X*_*t*_ is the input vector at time t; *h*_*t*_ is the state of the hidden layer at time t; *f*, *i*, *o*, are the forgotten gate, input gate and output gate, respectively; *C* is the state of the unit; *W*_*f*_,*W*_*i*_,*W*_*o*_,and*W*_*C*_ represent the corresponding weight coefficient matrices; *b*_*f*_,*b*_*i*_,*b*_*o*_,and*b*_*C*_ represent the corresponding bias values; σ(⋅) represents the sigmoid activation function; *tanh*⁡(⋅) represents the hyperbolic tangent activation function; ⊙ represents a dot multiplication operation.

Because of its special structure, LSTM not only has advantages in dealing with time series, but also can solve the problem of gradient vanishing in RNN. Generally, the information in an LSTM network is one-way transmission. BiLSTM combines forward and backward LSTM to obtain two hidden layers in different directions, thus obtaining more comprehensive information (Wang et al., 2019). The BiLSTM hidden layer states include the forward propagation states ${\vec{h}}_{t}$ and the backward propagation states ${\overset{\leftarrow }{h}}_{t}$ from _*H_t_*_ :


(6) $${\vec{h}}_{t}=\vec{L\,S\,T\,M}\,\left(h_{t-1},X_{t},C_{t-1}\right)\,t\in \left[1,n\right]$$

(7) $${\overset{\leftarrow }{h}}_{t}=\overset{\leftarrow }{L\,S\,T\,M}\,\left(h_{t-1},X_{t},C_{t-1}\right)\,t\in \left[n,1\right]$$

(8) $$H_{t}=\left[{\vec{h}}_{t},{\overset{\leftarrow }{h}}_{t}\right]$$

where *n* is the number of nodes in the hidden layer.

### Overall Framework of the Proposed Model

**FIGURE 3:**
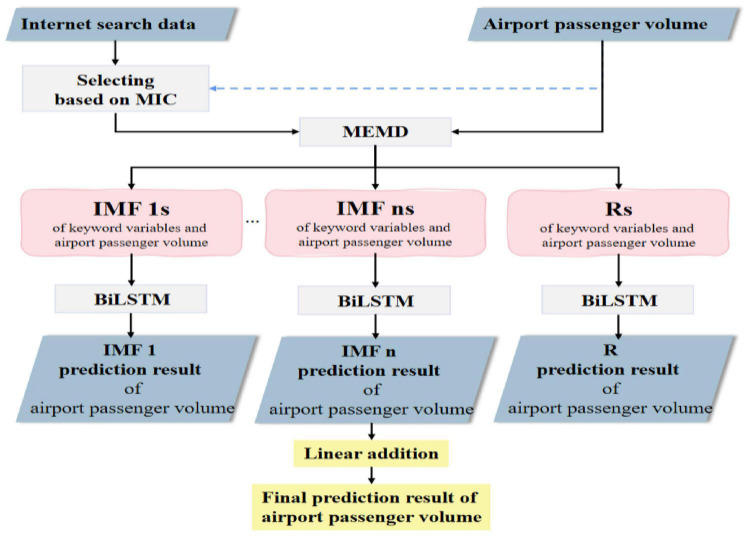
Structure of MIC_MEMD_BiLSTM prediction model.

Based on the influence of Internet search behavior on air passenger demand, combined with the basic principle of the above models, this manuscript presents a framework for air passenger demand forecasting. The framework makes full use of Internet search data and deep learning algorithms, which is noted as MIC_MEMD_BiLSTM. This framework takes a single airport as the research object, which is divided into two parts: data preprocessing and prediction, and its basic process is shown in Figure 3.

(1) Data preprocessing. Firstly, the keyword database is established by using relevant search recommendation and long tail keyword expansion method according to the core keywords. Next, the possible outlier values are identified and interpolated to get the initial search data. Then, in order to reduce the redundancy and avoid the influence of irrelevant variables on the prediction performance of the model, the Internet search data are filtered according to the maximum information coefficient (MIC) method (Reshef et al., 2011) to obtain the search data with high correlation to airport passenger demand. Compared with the traditional distance-based correlation measurement method, MIC method cannot only measure the linear and nonlinear relationship between variables in a large number of data, but also extensively mine the non-functional dependence relationship between variables, so the measurement of the interaction relationship between variables is more accurate. Finally, the data is divided into training set and test set.
(2) Prediction of airport passenger volume. MEMD is used to decompose airport passenger volume data and Internet search data simultaneously, and the corresponding sub-sequence groups are obtained. For each group of sub-sequences, BiLSTM model is established to predict the corresponding airport passenger volume sub-sequence. Finally, the prediction result of the airport passenger volume is obtained by summing the prediction results of each sub-sequence.

## Results

### Data Description

**FIGURE 4:**
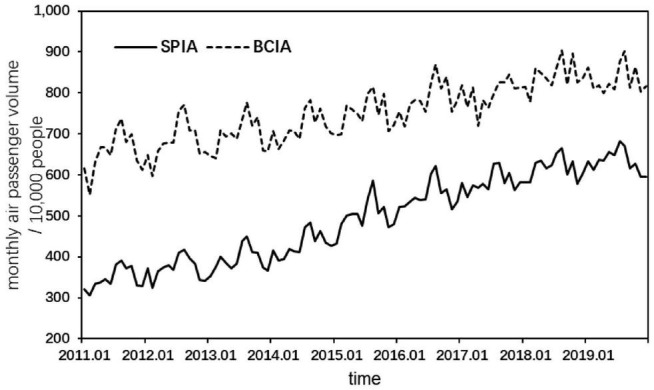
Air passenger volume of two airports from January 2011 to December 2019.

To verify the validity of the proposed prediction framework, Shanghai Pudong International Airport (SPIA) and Beijing Capital International Airport (BCIA) are selected as the research objects for empirical analysis. As shown in Figure 4, the highest data frequency available for the air passenger volume of the two airports is monthly, with each sample covering the period from January 2011 to December 2019 and containing 108 observations. The data were obtained from Wind database. For each sample, 96 observations from January 2011 to December 2018 are used as training set for model training. Twelve observations in 2019 are used as test set to test the prediction effect of the model.

A number of search engines are available on the Internet, where Baidu search engine has the highest user number and market share in China compared with other search engines (Xiang and Lu, 2019). In terms of Internet search data, as the research objects of this manuscript are Chinese airports, we adopt Baidu search index as Internet search data, which are taken from Baidu Index platform. In order to be consistent with the data frequency of the series to be predicted (air passenger volume), the daily data of keyword search volume from January 2011 to December 2019 were converted into monthly data.

### Processing and Screening of Internet Search Data

In this manuscript, the Internet search data used comes from the Baidu search index provided by Baidu Index platform, which is based on the search volume of Internet users in Baidu search engine and takes keywords as the statistical object. In other words, Baidu search index is the weighted sum of search frequency of each keyword in Baidu web search, which is obtained through scientific analysis and calculation. Taking the dataset of Shanghai Pudong International Airport as an example, the steps of obtaining and screening Internet search data in this manuscript are as follows:

#### Build a Word Library

First of all, several core keywords are screened according to the object to be predicted. Then we search these core keywords in Baidu search engine, and through relevant search recommendation and long tail keyword expansion method to expand the keywords, forming the corresponding keyword selection library. Finally, the keywords with less search volume will be removed. The keyword selection of Shanghai Pudong International Airport is shown in Table 1.

**TABLE 1:** Keyword selection of Shanghai Pudong International Airport.

No.	Keywords	No.	Keywords	No.	Keywords
1	Shanghai Pudong Airport	8	Shanghai Pudong Airport map	15	Pudong Airport bus schedule
2	Shanghai Pudong International Airport	9	Shanghai Pudong Airport parking fee	16	Pudong Airport duty-free shop
3	Shanghai Pudong Airport bus	10	Shanghai Pudong Airport duty-free shop	17	Pudong Airport parking fee
4	Shanghai Pudong Airport bus schedule	11	Shanghai Pudong Airport tel	18	Pudong Airport Terminal 2
5	Shanghai Pudong Airport to Hangzhou	12	Pudong International Airport	19	Pudong Airport to Hongqiao Airport
6	Shanghai Pudong Airport hotel	13	Pudong Airport	20	Pudong Airport to Shanghai Hongqiao Railway Station
7	Shanghai Pudong Airport near hotel	14	Pudong Airport flight enquiries		

#### Variable Selection

**FIGURE 5:**
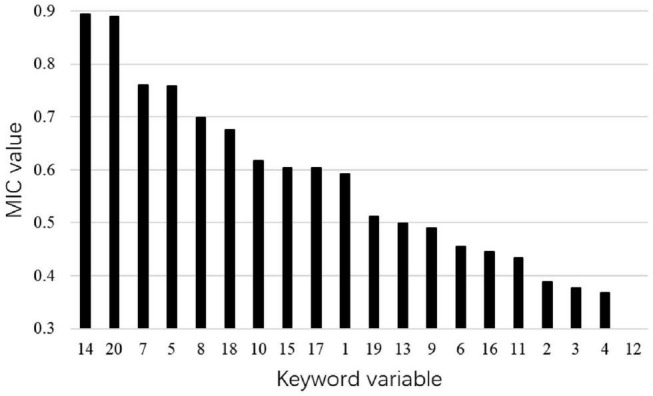
Maximum information coefficient (MIC) values between each keyword variable and passenger volume of Shanghai Pudong International Airport.

In order to obtain the keyword sequences with greater “contribution” to the forecast of airport passenger volume, we screen the keyword variables according to the MIC method. Figure 5 shows the MIC values between each keyword variable of Shanghai Pudong International Airport and its passenger volume. It can be seen that the MIC values of the first two keyword variables are significantly higher than that of other variables. For the purpose of reducing the computational complexity of the prediction model, only these two keyword variables are selected as exogenous input variables of the prediction model in the search engine data.

### Prediction Process

**FIGURE 6:**
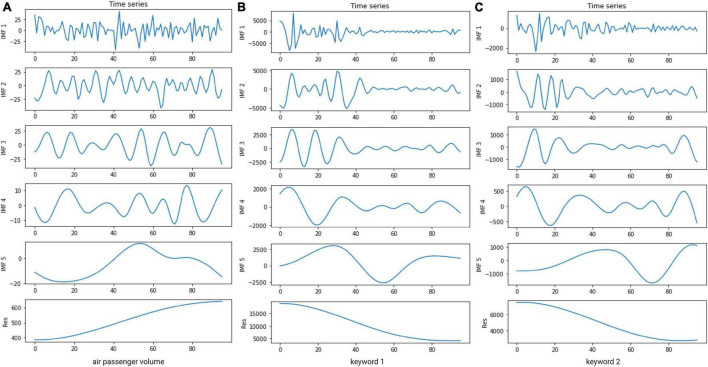
Decomposition results of air passenger volume of Shanghai Pudong International Airport and the corresponding two keyword sequences.

First, we use MEMD algorithm to decompose the airport passenger volume sequence and the selected keyword sequences simultaneously, so as to fully extract the data characteristics of each sequence. Figure 6 shows the sub-sequences of air passenger volume of Shanghai Pudong International Airport and the corresponding two keyword sequences obtained by MEMD decomposition. As can be seen from Figure 6, the three sequences are decomposed into the same number of IMF components, and the components with the same index have similar time scales. From IMF_1_ component to the residual sequence, the frequency of fluctuation gradually decreases, realizing pattern correspondence. Accordingly, the IMF components with the same index are formed into corresponding sub-sequence groups for the following prediction.

Then, for each sub-sequence group obtained in the previous step, a BiLSTM model is established to predict the corresponding sub-sequence of airport passenger volume. In the process of modeling, the input data includes historical data of airport passenger volume and the corresponding historical values of Internet search data, while the output data is only related to airport passenger volume. Because the air passenger volume data has a significant periodicity, with a 12-month cycle, showing similar changes every year, we set the input time dimension as 12 and the output time dimension as 3. That is to say, the historical data of the previous 12 months are used to predict the air passenger volume of the next 3 months.

Finally, the prediction results of all component sequences are summed up to obtain the final prediction result of airport passenger volume.

### Parameter Setting

In this manuscript, all models are implemented by Python software. Except for the BiLSTM model, other models are all adaptive models, which do not require parameter adjustment. The hyperparameters of the BiLSTM model are determined by the grid search method, and the parameter types and parameter selection are shown in Table 2.

**TABLE 2:** Hyperparameter selection of Bayesian bidirectional long short-term memory neural network (BiLSTM) model.

Parameter	Value
Activation function	{“sigmoid”, “tanh”, “relu”, “linear”}
Number of neurons in the hidden layer	6
Number of iterations	{500, 1,000, 1,500, 2,000}
Learning rate	{0.1, 0.01, 0.001}
Batch size	1/2 of the number of training samples
Objective function	Mean square error (MSE)
Optimization algorithm	Adam

### Prediction Performance Evaluation

#### Evaluation Index of Prediction Error

From the two perspectives of absolute error and relative error, we select three indicators, including root mean square error (RMSE), mean absolute error (MAE), and mean absolute percentage error (MAPE), to measure the prediction performance of the prediction model. The calculation formulas are as follows:


(9) $$\text{RMSE}=\sqrt{\frac{1}{n}\,\sum_{i=1}^{n}{\left(y_{i}-{\hat{y}}_{i}\right)}^{2}}$$

(10) $$\text{MAE}=\frac{1}{n}\,\sum_{i=1}^{n}\left|y_{i}-{\hat{y}}_{i}\right|$$

(11) $$\text{MAPE}=\frac{1}{n}\,\sum_{i=1}^{n}\left|\frac{y_{i}-{\hat{y}}_{i}}{y_{i}}\right|*100\%$$

where _*y_i_*_ and ${\hat{y}}_{i}$ represent the actual and predicted values of the original time series, respectively.

#### Diebold-Mariano Test

In this manuscript, we use Diebold-Mariano (DM) (Diebold and Mariano, 2002) statistic to compare the prediction performance of different models from statistical significance. The null hypothesis of the DM test is that there is no significant difference between the prediction performance of the test model (i.e., target model) and that of the comparison model (i.e., benchmark model). If the test result rejects the null hypothesis at a certain level of significance, it indicates that the test model is significantly better than the comparison model. Assuming that model A and model B are the two prediction models whose predictive effects need to be compared, the DM statistic can be defined as follows:


(12) $$S_{D\,M}=\frac{\bar{g}}{{\left({\overset{⌢}{V}}_{\bar{g}}/N\right)}^{1/2}}$$

where $\bar{g}=\frac{1}{N}\,\sum_{t=1}^{N}g_{t}$, $g_{t}={\left(x_{t}-{\overset{⌢}{x}}_{A,t}\right)}^{2}-{\left(x_{t}-{\overset{⌢}{x}}_{B,t}\right)}^{2}$, ${\overset{⌢}{V}}_{\bar{g}}=\gamma_{0}+2\sum_{l=1}^{\infty }\gamma_{l}$, and γ_*l*_ = *cov*(*g*_*t*_,*g*_*t*−*l*_); ${\overset{⌢}{x}}_{A,t}$ and ${\overset{⌢}{x}}_{B,t}$ are the predicted values of model A and model B at time t, respectively; *x*_*t*_ is the actual value of the original time series at time t; *cov*() represents an operator, which produces the covariance of two variables; N is the size of the test set. The DM statistic approximately follows the standard normal distribution. In this manuscript, the mean square error (MSE) is used as the loss function.

## Discussion

In this manuscript, in order to verify the effectiveness and superiority of the proposed model (i.e., model ➀ MIC_MEMD_BiLSTM), the comparison models are selected for experiments from the following three aspects:

(1) In order to verify that adding Internet search data can improve the accuracy of the prediction model, two sets of comparative experiments are set up, respectively. In the case of using the decomposition-ensemble strategy, the model that adds Internet search data (i.e., model ➀) is compared with the model that does not add Internet search data (i.e., model ➁ EEMD_BiLSTM). In the case of using a single prediction model, the model that adds Internet search data (i.e., model ➂ MIC_BiLSTM) is compared with the model that does not add Internet search data (i.e., model ➃ BiLSTM).
(2) In order to verify the rationality and effectiveness of the decomposition-ensemble strategy, two sets of comparative experiments are set up, respectively. In the case of adding Internet search data, the model using the decomposition-ensemble strategy (i.e., model ➀) is compared with the model using a single prediction model (i.e., model ➂). In the case of not adding Internet search data, the model using the decomposition-ensemble strategy (i.e., model ➁) is compared with the model using a single prediction model (i.e., model ➃).
(3) In order to further verify the comprehensive advantages of the proposed model that combines Internet search data, decomposition-ensemble strategy, and deep learning algorithm, we compare the proposed model with the deep learning method (i.e., model ➃ BiLSTM) and the traditional econometric models. Taking into account the strong periodicity of the data, in order to better demonstrate the advantages of the proposed model, two models ➄ SARIMA and ➅ HW, which are good at modeling seasonal data, are selected as the representative of the econometric models, and compared with the proposed model.

The prediction results of all the above models for airport passenger volume are shown in Table 3.

**TABLE 3:** Comparison of prediction results of all the models.

Model	SPIA			BCIA		
	MAE	MAPE (%)	RMSE	MAE	MAPE (%)	RMSE
➀ MIC_MEMD_BiLSTM	10.31	1.62	11.73	12.20	1.46	14.21
➁ EEMD_BiLSTM	11.93	1.88	14.20	13.60	1.61	17.06
➂ MIC_BiLSTM	11.56	1.84	12.77	14.41	1.71	21.22
➃ BiLSTM	13.92	2.21	15.52	20.18	2.41	24.46
➄ SARIMA	16.38	2.62	20.57	23.72	2.88	27.30
➅ HW	15.83	2.49	20.34	18.50	2.25	23.41

Based on the analysis of Table 3, the following conclusions can be drawn:

(1) In the case of using the decomposition-ensemble strategy, the prediction performance of the proposed model with Internet search data (i.e., model ➀) is better than that of the model without Internet search data (i.e., model ➁). For instance, the MAE of model ➀ was reduced by 15.7% compared to model ➁ on the SPIA dataset, and by 11.5% on the BCIA dataset. Meanwhile, in the case of using the single prediction model BiLSTM, the performance of the model with Internet search data (i.e., model ➂) is better than that of the model without Internet search data (i.e., model ➃). For example, the MAE of model ➂ was reduced by 20.4% compared to model ➃ on the SPIA dataset, and by 40.0% on the BCIA dataset. The comparison results show that adding Internet search data can improve the performance of air passenger demand forecasting model. Whether or not the data is decomposed, in either data set, scientifically filtered Internet search data can well compensate for the rigidity of structural historical statistics. It has been fully proved that Internet search data with reasonable selection and processing can reflect the travel intention of passengers, so as to express the corresponding demand for air passenger transport in advance.
(2) In the case of adding Internet search data, the prediction performance of the model using the decomposition-ensemble strategy (i.e., model ➀) is better than that of the model using a single prediction model (i.e., model ➂). For instance, the MAE of model ➀ was reduced by 12.1% compared to model ➂ on the SPIA dataset, and by 18.1% on the BCIA dataset. In the case of not adding Internet search data, the prediction performance of the model using the decomposition-ensemble strategy (i.e., model ➁) is also better than that of the model using a single prediction model (i.e., model ➃). For example, the MAE of model ➁ was reduced by 16.7% compared to model ➃ on the SPIA dataset, and by 48.4% on the BCIA dataset. The comparison results show that using the decomposition-ensemble strategy can effectively improve the prediction performance of the model. In other words, this strategy can indeed help the prediction model to capture and learn more effective information at different time scales, so as to improve the prediction accuracy.
(3) By comparing the prediction results of model ➀ with the deep learning algorithm (i.e., model ➃ BiLSTM) and traditional econometric models (i.e., model ➄ and ➅), it can be found that the prediction performance of model ➀ has been greatly improved, which reflects the comprehensive advantages of combining Internet search data, decomposition-ensemble strategy, and deep learning

algorithm. Moreover, in most cases, the prediction effects of traditional econometric models are worse than that of other comparison models. This is because the econometric models are good at describing the linear characteristics of the sequences, but for the sequences with nonlinear and non-stationary characteristics (such as the air passenger volume sequences), they have limitations in describing the characteristics of the sequences.

In this manuscript, in order to evaluate the superiority of the proposed model from a statistical point of view, the prediction results of all models are compared in pairs, and the corresponding DM statistics and p-values are calculated, respectively. The calculation results for SPIA and BCIA datasets are shown in Tables 4, 5, respectively. It can be seen from the tables that at a confidence level of 10%, the proposed model (i.e., model ➀) is significantly superior to most comparison models, indicating its statistical validity.

**TABLE 4:** Diebold-Mariano (DM) test results of different models for Shanghai Pudong International Airport (SPIA) dataset.

Target model	Benchmark model				
	➁ EEMD_BiLSTM	➂ MIC_BiLSTM	➃ BiLSTM	➄ SARIMA	➅ HW
➀ MIC_MEMD_BiLSTM	−0.9602 (0.1685)	−1.5926 (0.0556)	−1.5049 (0.0662)	−1.9787 (0.0239)	−1.3772 (0.0842)
➁ EEMD_BiLSTM		−0.0209 (0.4917)	−0.5669 (0.2854)	−1.2978 (0.0972)	−0.9495 (0.1712)
➂ MIC_BiLSTM			−0.4872 (0.3130)	−1.6397 (0.0505)	−1.0040 (0.1577)
➃ BiLSTM				−1.2994 (0.0969)	−0.8143 (0.2077)
➄ SARIMA					0.0613 (0.5244)

*The numbers in the table without ◯ are DM statistics, and the numbers in () are corresponding p-values.*

**TABLE 5:** Diebold-Mariano test results of different models for Beijing Capital International Airport (BCIA) dataset.

Target model	Benchmark model				
	➁ EEMD_BiLSTM	➂ MIC_BiLSTM	➃ BiLSTM	➄ SARIMA	➅ HW
➀ MIC_MEMD_BiLSTM	−08176 (0.2068)	−1.2720 (0.1017)	−1.9235 (0.0272)	−2.2517 (0.0122)	−1.5769 (0.0574)
➁ EEMD_BiLSTM		−0.7079 (0.2395)	−1.2356 (0.1083)	−1.7265 (0.0421)	−1.1504 (0.1250)
➂ MIC_BiLSTM			−1.6093 (0.0538)	−0.8516 (0.1972)	−0.4483 (0.3270)
➃ BiLSTM				−0.463 (0.3217)	−0.0422 (0.4832)
➄ SARIMA					1.5877 (0.9438)

*The numbers in the table without ◯ are DM statistics, and the numbers in () are corresponding p-values.*

Based on the above analysis, it can be seen that the proposed model (i.e., MIC_MEMD_BiLSTM) can obtain effective and stable prediction results, and has higher prediction accuracy than the benchmark models.

## Conclusion

From the perspective of broadening data sources and improving forecasting methods, this manuscript proposes an air passenger demand forecasting model that combines Internet search data, decomposition-ensemble strategy and deep learning algorithm. Based on the empirical analysis of Shanghai Pudong International Airport and Beijing Capital International Airport, it can be found that because of the addition of Internet search data as a new form of input data, and the advantages of decomposition-ensemble strategy and deep learning algorithm, the proposed model can fully extract the complex features of the original time series with different scales, and obtain more accurate and reliable prediction results than the benchmark models. This also further illustrates that adding appropriate Internet search data into the air passenger demand forecasting model can effectively improve the forecasting accuracy.

In addition, although the proposed model has achieved good prediction results, it still has some limitations. For example, the proposed model only includes Baidu search index as a representative of Internet search data. In future research, other types of Internet data (such as Google Trends) and other air passenger demand impact factors (such as GDP) can also be considered to enrich the number of input variables for the prediction model.

## Data Availability Statement

Publicly available datasets were analyzed in this study. This data can be found here: https://github.com/QingZ96/Air-Passenger-Demand-Forecasting.

## Author Contributions

XL devised the study and collected the data. QZ and CH analyzed the data. MY wrote the manuscript. WN helped with writing and editing the manuscript. All authors contributed to the article and approved the submitted version.

## Conflict of Interest

The authors declare that the research was conducted in the absence of any commercial or financial relationships that could be construed as a potential conflict of interest.

## Publisher’s Note

All claims expressed in this article are solely those of the authors and do not necessarily represent those of their affiliated organizations, or those of the publisher, the editors and the reviewers. Any product that may be evaluated in this article, or claim that may be made by its manufacturer, is not guaranteed or endorsed by the publisher.
